# Parathyroid Hormone on Osteoprotegerin Levels in Patients with Primary Hyperparathyroidism

**DOI:** 10.5152/eurasianjmed.2021.21197

**Published:** 2022-10-01

**Authors:** Berna Okudan, Bedri Seven, Alper Çağrı Karcı, Muhammed Fevzi Kılınçkaya, Mustafa Çapraz, Turan Turhan, Nedim Cüneyt Murat Gülaldı

**Affiliations:** 1 Department of Nuclear Medicine, Ankara City Hospital, University of Health Sciences, Ankara, Turkey; 2 Department of Nuclear Medicine, Sabuncuoğlu Şerefeddin Training and Research Hospital, Amasya, Turkey; 3 Division of Endocrinology, Department of Internal Medicine, Ankara City Hospital, Ankara, Turkey; 4 Department of Biochemistry, Ankara City Hospital, Ankara, Turkey; 5 Department of Internal Medicine, University of Amasya Faculty of Medicine, Amasya, Turkey; 6 Department of Nuclear Medicine, Ankara City Hospital, Ankara, Turkey

**Keywords:** Bone mineral density, osteoprotegerin, parathyroid hormone, primary hyperparathyroidism

## Abstract

**Objective:**

Osteoprotegerin is a glycoprotein that plays a major role in the regulation of bone turnover. The influence of parathyroid hormone, an important regulator of bone remodeling, on osteoprotegerin production is controversial. The purpose of the study was to assess the influence of parathyroid hormone on the circulating level of osteoprotegerin in patients with primary hyperparathyroidism by comparing it with healthy controls.

**Materials and Methods:**

Forty-four patients with biochemical verification of primary hyperparathyroidism scheduled for the surgical cure and 38 healthy subjects were included. Blood samples of the study group were taken before surgery. Levels of serum parathyroid hormone, osteoprotegerin, calcium, 25-hydroxyvitamin D [25(OH)D], and alkaline phosphatase were analyzed. Bone mineral density at the L1-L4 vertebrae and femoral neck was calculated by dual-energy X-ray absorptiometry.

**Results:**

Osteoprotegerin levels and bone mineral density values were significantly lower in patients than in the healthy subjects (*P* = .002 and *P* < .0001, respectively). There was no correlation between osteoprotegerin and parathyroid hormone in the groups. Osteoprotegerin was weakly correlated with bone mineral density in patients. No correlation was noted between osteoprotegerin and bone mineral density in the control group. Furthermore, osteoprotegerin levels were not correlated with calcium, 25(OH)D, and alkaline phosphatase levels in each group.

**Conclusion:**

The production of osteoprotegerin appears to be inhibited by parathyroid hormone in patients with primary hyperparathyroidism. A weak positive correlation found among osteoprotegerin and bone mineral density recommends that osteoprotegerin may be a molecule that impacts bone metabolism and finally bone mineral density.

**Main Points**

- The expression of osteoprotegerin (OPG), which plays an important role in the bone remodeling process, is regulated by many hormones, one of the most important being parathyroid hormone (PTH).
- In our study, serum OPG levels and mean bone mineral density (BMD) values were lower in patients with primary hyperparathyroidism than in the healthy control group. Furthermore, there was a weak positive correlation between OPG and BMD.
- The results of our study suggest that OPG production may be inhibited by PTH and that OPG may be a molecule that affects bone metabolism and ultimately BMD.

## Introduction

Bone is an active tissue that is continually renewed to maintain durability and entirety. This complex process, called bone remodeling, involves the resorption of bone and subsequent formation of bone by osteoclasts and osteoblasts, respectively. Some glycoproteins such as osteoprotegerin (OPG) have very important roles during the bone remodeling process.

Osteoprotegerin is a soluble molecule of the tumor necrosis factor receptor group secreted by various tissues and cell types, together with the osteoblast lineage cells.^1,2^ It inhibits osteoclastogenesis by blocking the maturation of osteoclasts, thus playing a crucial role in the modulation of cell function, differentiation, and survival during the bone remodeling process.^3-8^ The expression of OPG is managed by various hormones and cytokines, where parathyroid hormone (PTH) is one of the very important hormones, and forms the basis of the pathology of various skeletal diseases with increased bone loss/formation or disorders with changed bone remodeling.^9-12^

Primary hyperparathyroidism (PHPT), a common endocrine disturbance, is defined by hypercalcemia caused by excessive uncontrolled secretion of PTH from the parathyroid glands.^13,14^ Parathyroid hormone is a considerable organizer of bone remodeling, the process of renewal of the skeleton that occurs continuously throughout life.^15^ Hypersecretion of PTH results in a rise in bone turnover, mostly in resorption, leading to a raised risk of fracture.^2,14^

The purpose of our study is to assess the impact of PHPT on the circulating level of OPG by comparing it with healthy volunteers.

## Materials and Methods

### Patients

The study was conducted with 44 patients (13 males and 31 females, mean age 53.04 ± 10.01 years) with PHPT, who were candidates for surgical therapy. The diagnosis of PHPT was made based on laboratory analyses characterized by high PTH and calcium levels. Furthermore, postoperative histopathological reports confirmed the presence of parathyroid adenoma as the cause of PHPT for all patients. The laboratory workup, dual-phase technetium-99m-methoxyisobutylisonitrile (^99m^Tc-MIBI) single-photon emission computed tomography/computed tomography (SPECT/CT) imaging, which enhances the surgeon’s success with accurate anatomic localization of the abnormal parathyroid gland(s), and bone mineral density (BMD) analysis were performed in all patients. Patients with other disorders and receiving drugs well-known to influence bone or mineral metabolism, and those with impaired kidney and liver functions and with a smoking habit were excluded from the study. Thirty-eight healthy volunteers (10 males and 28 females, mean age 51.13 ± 8.4 years) who had no history of bone disorders, did not use drugs that could influence bone density and PTH or calcium levels, and were not smokers were included as the control group. Ethical committee approval was received from the Ethics Committee of Ankara Numune Training and Research Hospital, (Approval no: 20796219-1011/2015). Written informed consent was obtained from all participants who participated in this study.

### Biochemical Analysis

Preoperative blood samples were taken after 1 night of fasting. Levels of PTH, calcium, and alkaline phosphatase were calculated by an AU5800 automated chemistry analyzer (Beckman Coulter, Brea, Calif, USA). Levels of 25-hydroxyvitamin D [25(OH)D] were measured using an electroluminescence method on the Cobas E-411 analyzer (Roche Diagnostics GmbH, Mannheim, Germany). Serum OPG levels were calculated by a commercially available enzyme-linked immunosorbent assay (ELISA) kit with respect to the directives supplied by the producer (eBioscience Inc., San Diego, Calif, USA).

### Bone Mineral Density

Calculations of BMD at the L1-L4 vertebrae and femoral neck were performed by dual-energy X-ray absorptiometry (Hologic QDR-4500, Hologic Inc., Bedford, Mass, USA). T- and Z-scores were measured regarding a regional regular society BMD database. T-score less than −2.5 was expressed as osteoporosis and a T-score between −1 and −2.5 was expressed as osteopenia, in accordance with the World Health Organization criteria.

### ^99m^Tc-MIBI SPECT/CT Imaging

Dual-phase parathyroid planar screenings in the cervical region were acquired at 15 minutes and 150 minutes following intravenous administration of 555 MBq ^99m^Tc-MIBI by a low-energy high-resolution hybrid SPECT/CT scanner (GE Infinia Hawkeye 4, GE Healthcare, Buckinghamshire, UK) collimators. Images were acquired using a 256 × 256 matrix. The energy window of 20% at 140 keV photopeak was chosen. Single-photon emission computed tomography/computed tomography was carried out promptly following the delayed parathyroid screening.

### Statistical Analysis

Statistical investigation was carried out by SPSS version 23.0 (IBM Corp., Armonk, NY, USA) and a *P*-value of less than .05 was accepted as statistically significant, with information in the document presented as median (interquartile range). Correlations were evaluated by Spearman’s rank correlation coefficient. Differences among the 2 groups were evaluated by the Mann–Whitney *U* test.

## Results

Table 1 shows serum biochemical parameters and BMD of participants with histopathologically confirmed parathyroid adenoma whose SPECT/CT images were determined to be truly positive in the study. All patients had raised PTH and calcium levels over the reference values, and the mean serum concentration of alkaline phosphatase (ALP) was increased. Also, 25(OH)D levels were slightly higher in patients than in controls. The serum concentration of OPG was lower in patients with PHPT than in the healthy subjects. The PHPT patients were usually osteoporotic, and mean BMD values were significantly lower in patients than in the healthy subjects.

**Figure 1.**
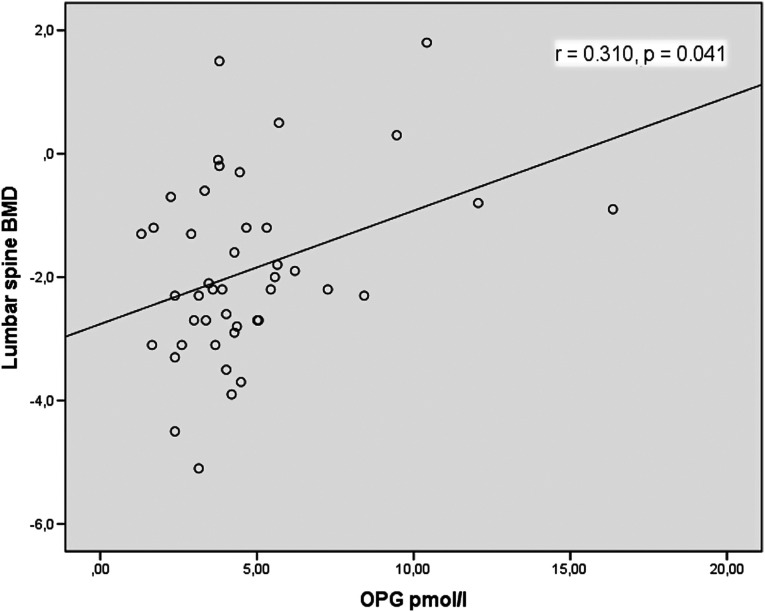
Correlation between lumbar spine BMD and OPG in PHPT patients. BMD, bone mineral density; OPG, osteoprotegerin; PHPT, primary hyperparathyroidism.

**Figure 2.**
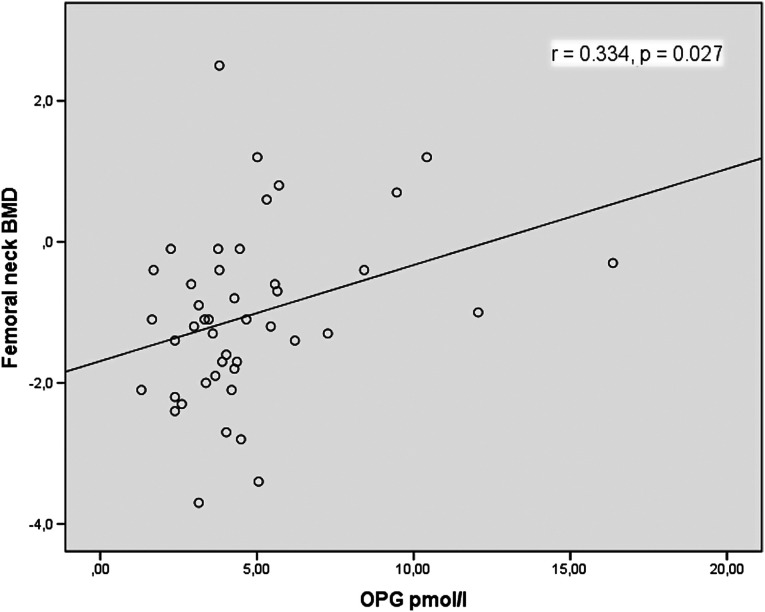
Correlation between femoral neck BMD and OPG in PHPT patients. BMD, bone mineral density; OPG, osteoprotegerin; PHPT, primary hyperparathyroidism.

There was no correlation among serum OPG and PTH in the PHPT patients’ group (*r* = 0.037, *P* = .812) as well as in the healthy controls (*r* = −0.075, *P* = .654). Serum OPG was weakly correlated with BMD in PHPT patients (as shown in Figure 1, at the spine, *r* = 0.310, *P* = .041; as shown in Figure 2, at the femoral neck, *r* = 0.334, *P* = .027). On the other hand, no correlation was noted between serum OPG and BMD in the control group. Furthermore, OPG levels were not correlated with calcium, 25(OH)D, and ALP levels in each group.

## Discussion

Following the discovery of OPG as one of the main factors in managing osteoclast formation and activation, numerous studies have been conducted to research the role of this molecule in bone metabolism and its contribution to the formation of various bone diseases. However, despite significant research efforts, the influence of PTH on producing OPG is arguable.

Previous in vitro studies demonstrated that PTH reduces OPG gene expression in osteoblasts.^16,17^ In humans, serum OPG did not alter in postmenopausal women with osteoporosis after intermittent PTH 1–34 administration.^18^ In another study, it was stated that serum OPG did not appear to be affected in patients with PHPT.^19^ Two descriptive, cross-sectional researches addressed the correlation between serum OPG and PTH in healthy individuals. One was 242 men between 19 and 85 years of age and demonstrated a negative correlation between PTH and serum OPG.^20^ However, a weak positive correlation was shown in another study of 490 women over 65 years of age.^21^ One study reported that there was no correlation between PTH and OPG and that OPG levels were higher in PHPT patients than in the healthy subjects.^11^

In our study, the serum concentration of OPG in patients with PHPT was lower than in the control group. Also, no correlation was found between serum OPG and PTH in each group. We think this may stem from long-standing uplifts in the PTH, which may perhaps lead to longtime declines in cellular capability for OPG production, as seen in the PHPT.

The correlation between bone markers and serum OPG differs similarly among researches. One study demonstrated that elevated ALP was associated with elevated serum OPG levels,^22^ while others reported a reverse relation between serum osteocalcin and OPG.^22^ Still, others were not found.^20^ Our study showed that there was no correlation between serum OPG levels and bone markers such as ALP and calcium.

Clinical observation researches on the relation between serum OPG and BMD are uncertain. In spite of favorable impacts of OPG as an antiresorptive agent in clinical investigations,^23^ a study demonstrated that an elevated serum OPG level along with low BMD.^21^ On the other hand, other studies have not reported this correlation.^20,21^ In this study, although low BMD values were observed in PHPT patients, a poor positive correlation was detected among OPG levels and BMD. No correlation was found among OPG levels and BMD in the control group.

One of the limitations of the study is that the majority of patients were females, thus the results may not be generalizable. Another limitation is that serum concentration of OPG is regulated by various tissues and thus as a result, serum levels might only partly indicate the real regional bone microenvironment.

In conclusion, serum OPG levels and mean BMD values were low in patients with PHPT, and there was a poor positive correlation bewteen OPG and BMD, suggesting that OPG production may be inhibited by PTH and that OPG may be a molecule that affects bone metabolism and ultimately BMD. Further research is required to support these findings.

## Figures and Tables

**Table 1.** Biochemical Parameters and BMD of Participants

		PHPT Patients (n = 44)	Control Group (n = 38)	*P*
OPG	pmol/L	4.02 (1.31-16.36)	5.77 (1.35-10.33)	.002
PTH	pg/mL	175.50 (81.00-761.00)	51.50 (17.00-89.00)	<.0001
Calcium	mg/dL	11.10 (9.86-13.80)	9.40 (8.40-10.10)	<.0001
25(OH)D	ng/mL	14.90 (5.00-32.00)	12.00 (6.40-31.00)	.115
ALP	U/L	107.00 (43.00-1080.00)	76.50 (44.00-116.00)	<.0001
BMD				
Spine		−2.20 (−5.10 to 1.80)	0.70 (−1.20 to 2.10)	<.0001
Femoral neck		−1.10 (−3.70 to 2.50)	0.65 (−1.50 to 2.20)	<.0001

Values are given as median (interquartile range). *P*-values are from the Mann–Whitney *U* test.

OPG, osteoprotegerin; PTH, parathyroid hormone; 25(OH)D, 25-hydroxyvitamin D; ALP, alkaline phosphatase; BMD, bone mineral density.
